# In-Brain Multiphoton Imaging of Vaterite Cargoes Loaded with Carbon Dots

**DOI:** 10.1021/acs.nanolett.4c00325

**Published:** 2024-05-23

**Authors:** Hani Barhum, Cormac McDonnell, Oleksii Peltek, Rudhvi Jain, Mariam Amer, David Kain, Galit Elad-Sfadia, Muhammad Athamna, Pablo Blinder, Pavel Ginzburg

**Affiliations:** † Department of Electrical Engineering, Tel Aviv University, Ramat Aviv, Tel Aviv 69978, Israel; ‡ Triangle Regional Research and Development Center, Kfar Qara 3007500, Israel; § Light-Matter Interaction Centre, Tel Aviv University, Tel Aviv 69978, Israel; ∥ School of Physics and Engineering, ITMO University, St. Petersburg 191002, Russian Federation; ⊥ Neurobiology, Biochemistry and Biophysics School, Wise Life Science Faculty, Tel Aviv University, Tel Aviv 69978, Israel; # Sagol School of Neuroscience, Tel Aviv University, Tel Aviv 69978, Israel

**Keywords:** Two-photon fluorescence (2PF), Cross-section, Phenylenediamine, Bioimaging, Vaterite, Carbon Dots

## Abstract

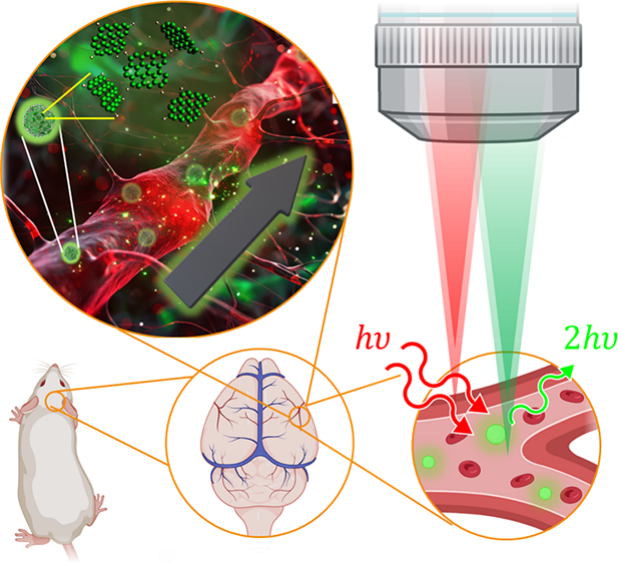

Biocompatible fluorescent agents are key contributors to the theranostic paradigm by enabling real-time in vivo imaging. This study explores the optical properties of phenylenediamine carbon dots (CDs) and demonstrates their potential for fluorescence imaging in cells and brain blood vessels. The nonlinear absorption cross-section of the CDs was measured and achieved values near 50 Goeppert-Mayer (GM) units with efficient excitation in the 775–895 nm spectral range. Mesoporous vaterite nanoparticles were loaded with CDs to examine the possibility of a biocompatible imaging platform. Efficient one- and two-photon imaging of the CD–vaterite composites uptaken by diverse cells was demonstrated. For an in vivo scenario, CD–vaterite composites were injected into the bloodstream of a mouse, and their flow was monitored within the blood vessels of the brain through a cranial window. These results show the potential of the platform for high-brightness biocompatible imaging with the potential for both sensing and simultaneous drug delivery.

Biocompatible fluorescent agents, such as carbon dots (CDs), have become a cornerstone in theranostics by enabling real-time in vivo imaging of drug delivery cargoes.^1,2^ CDs have attracted considerable attention across various disciplines, including bioimaging,^3^ biosensing,^4^ and biotherapy,^5,6^ because of their numerous advantages. CDs can be synthesized through facile production routes in wet chemistry and physical processes,^7^ including laser ablation,^8^ microwave-assisted methods,^9^ and plasma processing.^10^ Their physicochemical properties, including tunable fluorescence^11^ and high quantum yield,^12^ are related to their carbon source^13^ and synthesis conditions,^14^ such as reaction temperature,^15^ time,^16^ pH,^17^ and postsynthetic treatments.^18^

The potential biocompatibility of CDs compared with common toxic inorganic crystalline quantum dots^19^ and other organic dyes^20,21^ places them at the forefront of bioimaging and biosensing agents.^22^ Moreover, CDs can be modified to act as targeted binders.^23,24^ Thus, they can be engineered to recognize and bind to specific sites on cells or tissues, thereby enhancing their utility in targeted imaging.^25^ This also makes the site visible or trackable, which is particularly useful in the real-time monitoring of biological processes.^26^ Moreover, several types of CDs exhibit outstanding nonlinear responses in multiphoton fluorescence (MPF).^27^ Therefore, their potential for use in deep-tissue imaging and therapy has been the subject of several studies.^28,29^ For example, nitrogen-doped CDs have been developed for high-resolution imaging and ultrasensitive sensing of metal ions,^30^ while two-photon radiometric carbon dot-based nanoprobes have been used for real-time monitoring of intracellular pH.^31^ Polyphenolic carbon quantum dots are also employed for membrane-targeting and drug delivery in tumor therapy.^32^ Furthermore, the nonlinear response of CDs can be exploited for photodynamic therapy^33^ where the energy from the absorbed photons is used to generate reactive oxygen species to kill cancer cells. Multicolored phenylenediamine CDs have diverse applications;^30,34^ these colorful, bright CDs show imaging, sensing, and therapy abilities, which makes them particularly attractive for further theranostic development.

Theranostics combines diagnostics and therapy in a single platform, which necessitates drug delivery nanocapsules to possess multiple functions, including trackability with high spatial resolution.^35^ Notably, theranostic applications of CDs are usually realized in combination with other materials.^36^ In this context, mesoporous calcium carbonate (CaCO_3_) in vaterite form has emerged as an exceptionally promising load-carrying particle. This non-organic nanoparticle offers several advantages, including a high load capacity, large surface area, as well as facile chemistry for ligand binding, which allows it to accommodate different types of cargo.^37−39^ Furthermore, its biodegradability makes it a safe option for in vivo applications.^40,41^ The potential integration of CDs with vaterite nanoparticles combines the imaging capabilities of CDs with the drug delivery potential of vaterite to create a powerful platform for theranostics. Finally, despite considerable research efforts dedicated to developing nanoparticle-based drug delivery systems for cancer treatment, the efficiency of nanoparticle accumulation at the tumor site often falls short and rarely exceeds a few percent.^42^ This limitation underscores the importance of in vivo studies to optimize nanoparticle properties for a more efficient drug delivery process.^43^

Here, we explore multiphoton imaging of vaterite nanoparticles loaded with phenylenediamine CDs, as shown in Figure 1. The imaging ability of the CD–vaterite composite is demonstrated through single and multiphoton fluorescence microscopy of cells and in vivo brain vessels. The CDs are characterized by several methods, including Fourier transform infrared (FTIR) spectroscopy, X-ray photoelectron spectroscopy, and transmission electron microscopy (TEM). The two-photon absorption cross-section of phenylenediamine CDs in biological conditions was characterized and compared with fluorescein. The CDs were then loaded onto vaterite nanoparticles and used for one- and two-photon imaging in vitro. Finally, the CD–vaterite composites were imaged in vivo in brain vessels to provide a powerful tool for studying drug–cell interactions. As an outlook, the demonstrated approach contributes to developing multifunctional optical tools for drug–cell interaction studies.

**Figure 1:**
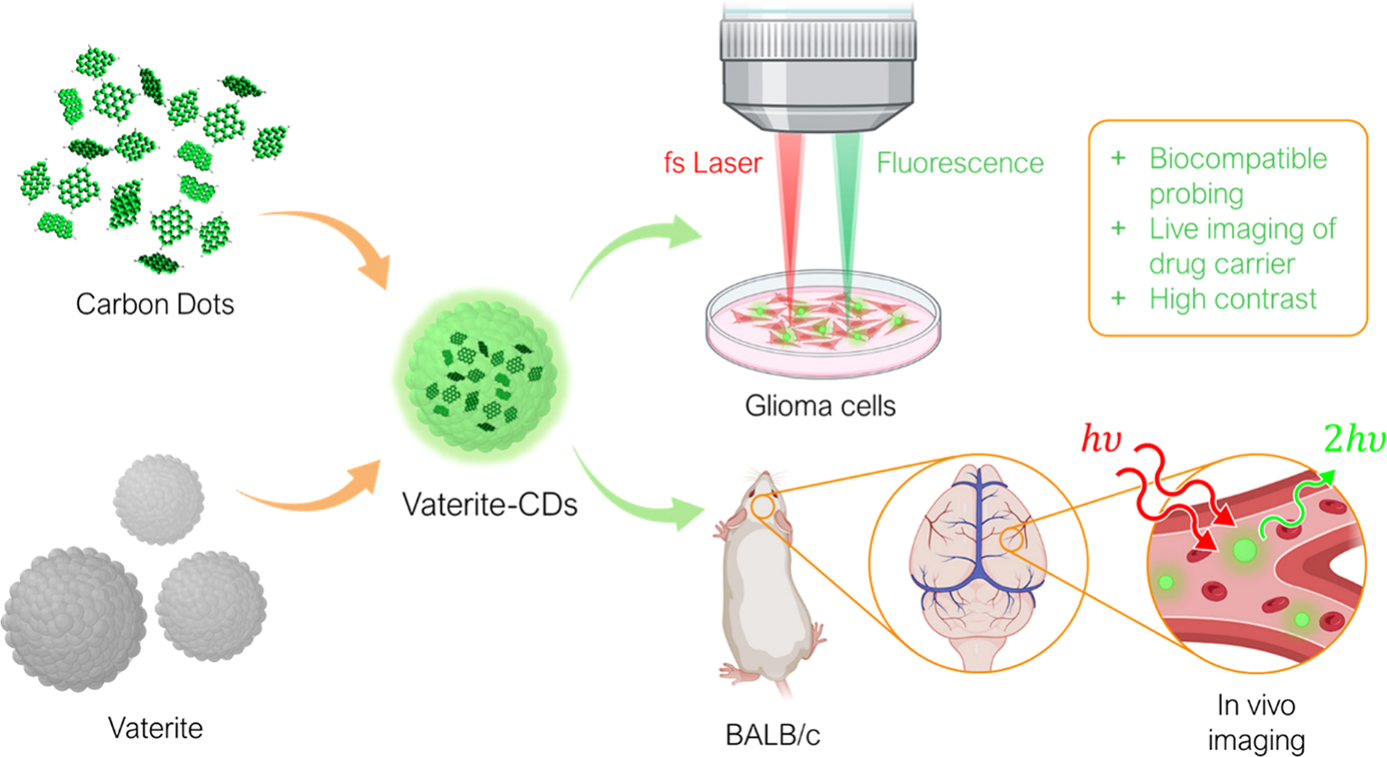
Experimental concept. (Left) CD synthesis followed by mesoporous vaterite encapsulation. (Middle) Vaterite particles loaded with CDs. (Top right) Illustration of glioma cells incubated with fluorescent particles excited with a NIR femtosecond laser. (Bottom right) Illustration of in vivo imaging of CD–vaterite composites circulating in the brain vasculature.

Phenylenediamine, characterized by its two amine groups, plays a crucial role during the carbonization process by acting as a hotspot. The growth of the CDs is regulated on two levels. First, the solvent ethylene glycol constrains the diffusion of reactive species. Second, adding an acid or proton source (in this case 1%) shields the hot spot, which results in kinetic control over the final product. This dual-level regulation directly influences the quantity of the product, its band gap, and the fluorescence quantum efficiency^30^ (see the Supporting Information for CD synthesis protocol).

In this study, carbon dots (CDs) with a diameter of approximately 3–5 nm were meticulously characterized to confirm their structural and chemical properties, with full details presented in the Supporting Information (Figure S1). Transmission electron microscopy (TEM) illustrated the uniform size distribution of the CDs, which is critical for their effective encapsulation within mesoporous particle volumes. Comprehensive spectroscopic analyses, including Fourier transform infrared (FTIR) and X-ray photoelectron spectroscopy (XPS), revealed various functional groups that endow the CDs with their unique fluorescence and chemical binding capabilities. The FTIR spectrum highlighted groups essential for specific site imaging, such as −CH_2_, aromatic −CH bonds, and carboxylic groups, while the XPS analysis provided insights into the CDs’ versatile surface chemistry, which enhances their dispersibility and stability in biological environments. These detailed characterizations are presented in detail in the Supporting Information.

The linear and nonlinear optical properties of the synthesized CDs were examined. A description of the optical setups can be found in the Supporting Information. First, Figure 2a illustrates the CDs’ UV–VIS absorbance spectrum. The CDs exhibit two main absorbance bands; the first band at 450 nm results from surface states and surface oxidation. The UV band around 270 nm arises from the π → π* and n → π* transitions associated with conjugated C=C and C=O bonds. An emission band is observed at 525 nm.

**Figure 2:**
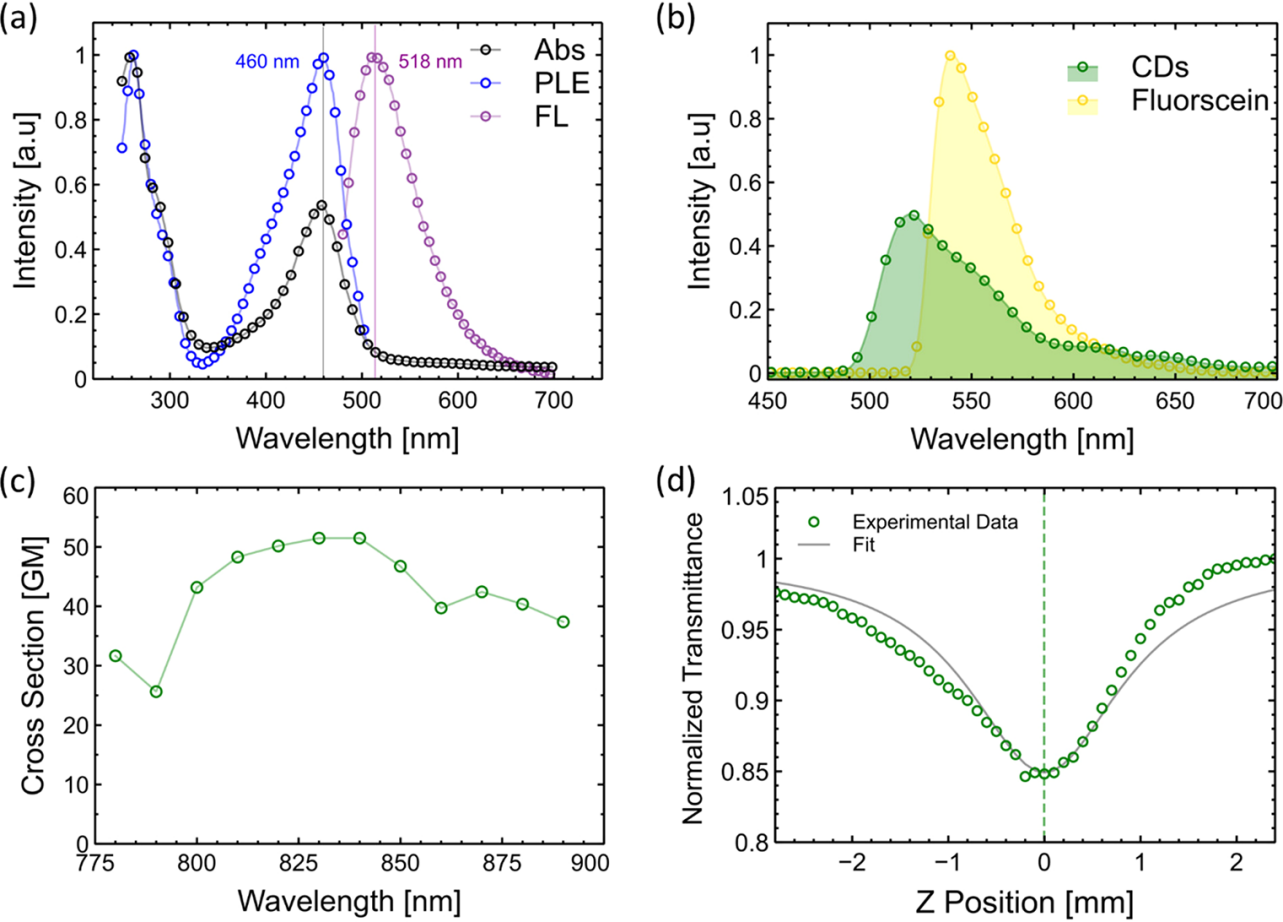
Linear and nonlinear optical properties of CDs. (a) Single-photon absorbance (represented by black circles), single-photon luminescence excitation (blue) monitored at 530 nm, and single-photon fluorescence (green) under 450 nm excitation spectra. (b) Two-photon fluorescence spectra of CDs (represented by green circles) and fluorescein (yellow) under 800 nm femtosecond pump irradiation. The relative peak strength is normalized to 1. (c) Two-photon absorption cross-section of CDs (calibrated to fluorescein) versus pump wavelength. (d) Open aperture Z-scan of CDs (0.03 mg/mL, in an ethanol solution) in a 2 mm quartz cuvette.

The two-photon fluorescence spectra of the CDs and fluorescein are shown in Figure 2b following excitation with an 800 nm femtosecond pump. The emission band of the CDs is broader than fluorescein and extends from green 490 to 600 nm with a lower intensity up to 650 nm. The normalized intensity across the CDs spectrum, which has a quantum yield (QY) of 35%, is approximately 0.6 when compared with fluorescein, which has a QY of 91%. The two-photon cross section was calculated relative to fluorescein, which served as a reference standard. The method for the cross-section calculation is described in the Supporting Information. This calculation was performed in the wavelength range of 780–900 nm, and the results are shown in Figure 2c. The cross-section in Goeppert-Mayer (GM) units is approximately 30 at 780 nm excitation and above 50 GM at 830 nm. Beyond this point, the value decreases to 50 GM and oscillates between 50 and 30 GM with increasing wavelength. For comparison, the fluorescein cross section is 40 GM around wavelengths of 800 nm. The nonlinear two-photon absorbance of the CDs was measured using the open-aperture Z-scan method (see the Supporting Information for method and fitting information).^44^ An average laser power of 120 mW illuminated the sample with a CD concentration of 0.01 mg/mL in ethanol. The solution was placed in a 2 mm thick quartz cuvette (Figure 2d). The CD’s two-photon absorption coefficient is estimated to be 2 cm W^–1^ from the fit. In comparison, several other materials, including organic molecules like coumarin,^45^ bis(styryl)benzene derivatives,^46^ and quantum dots,^47^ exhibit extremely high absorption coefficients ranging from 100 to 10 000 GM. Finally, a Z-scan of vaterite without any CDs showed no two-photon absorption.

In the next phase, the CDs were encapsulated within vaterite nanoparticles with different geometries. We fabricated vaterite cargo particles of varying geometries and immersed them in CD solutions of different concentrations. The SEM images in Figure 3a–c show the precise geometry (spherical, elliptical, and toroidal) and inherent porosity of the vaterite particles before CD encapsulation. The CD–vaterite composites were then imaged using a confocal microscope and measured to be approximately 500 nm for individual particles of all three geometries. The volume across all three geometries was chosen to be near constant. More statistical info from the SEM data and confocal images is presented in the Supporting Information. Selected confocal images of each geometry are presented in Figure 3d–f. Although the resolution was not high enough to determine the exact concentration of the CDs within the volume, a general trend could be identified. In general, the toroidal particles exhibited greater brightness, which suggests a higher presence of fluorescent agents on the surface since there is no significant loading of CDs relative to other shapes. The fluorescent image of the toroidal particle also mirrors its donut-shaped topology. Data from 10 particles were collected, and the average distributions as displayed in the panels with error bars represented by vertical lines, as shown in the Supporting Information Figure S2. The fraction of CDs adsorbed on the vaterite was quantified using the Langmuir parameters and loading properties. Figure 3g shows the Langmuir adsorption curves of CD molecules on the vaterite surface plotted against the CD concentration in the solution during fabrication. The adsorption reached saturation between 0.7–0.9 mg g^–1^ with elliptical particles achieving the highest CD load capacity. The linearization is described in the Supporting Information. Equation S1 of the Langmuir equation enabled the extraction of the isotherm parameters seen in Table 1. In general, despite the differences in the shape properties, the number of CDs loaded on a particle is only weakly dependent on its shape, as shown in Figure 3h. The Langmuir adsorption parameters further show this observation. The Langmuir constant *K*, which reflects the affinity between the CDs and the particles, remains relatively consistent across different particle geometries. This suggests that the inherent binding energy or adhesion energy between the CDs and the particles is similar, irrespective of the particle’s shape. However, the maximum adsorption capacity *q*_m_ does exhibit variations, and with elliptical particles, there is a slightly higher capacity. This could be attributed to the unique surface properties or internal structure of the elliptical particles, which might offer more favorable sites for CD adsorption. Furthermore, it is worth noting that the vaterite surface can be modified to increase its affinity to CDs, for instance by polymer addition.

**Table 1:** Langmuir Isotherm Parameters for Different Shapes of Vaterite

geometry		
spherical	0.8	61.98
elliptical	0.85	62.99
toroidal	0.7	63.74

**Figure 3:**
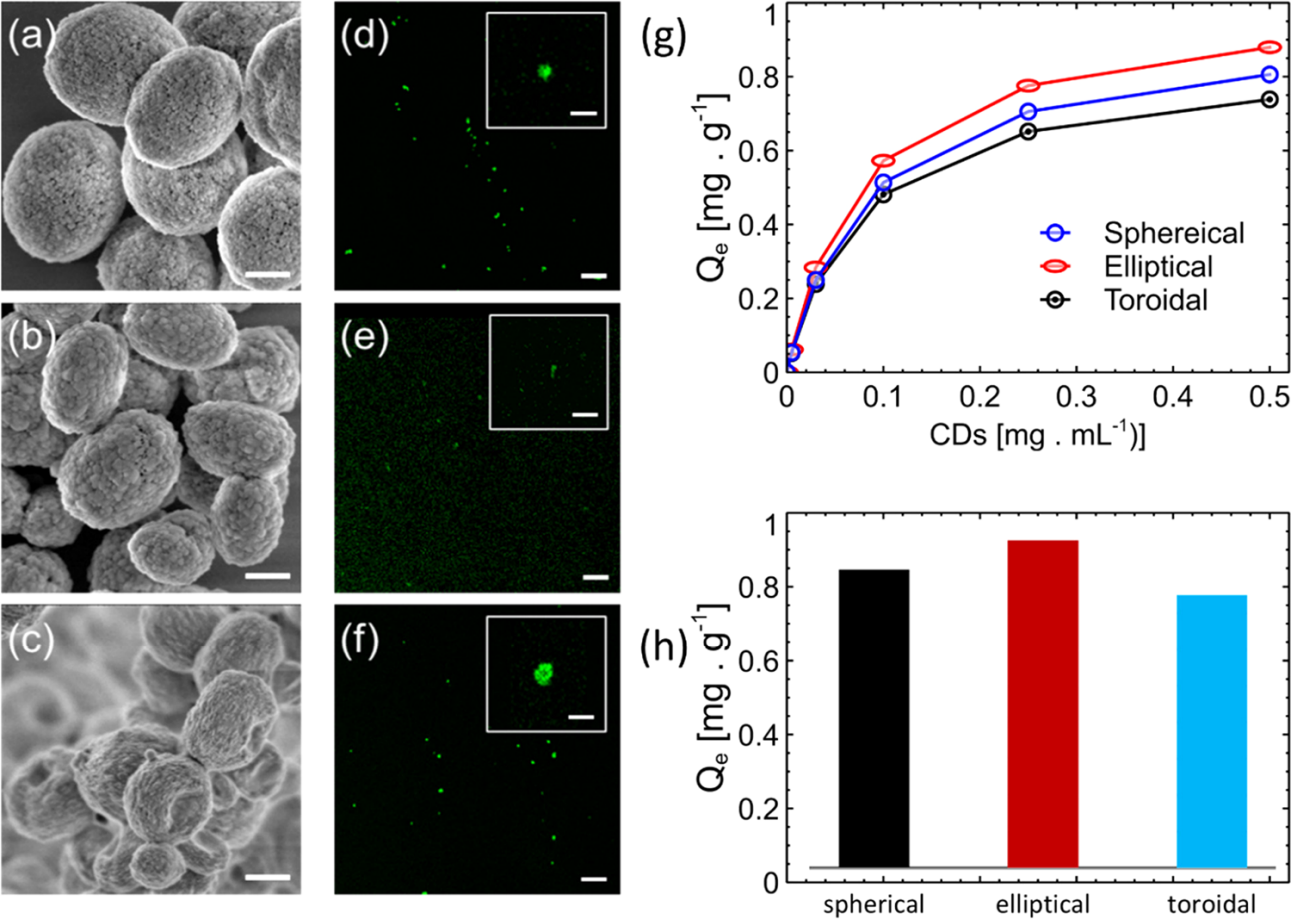
(a–c) Scanning electron microscope images of vaterite particles in three different geometries: (a) spheres, (b) ellipsoids, and (c) toroids. The scale bar is 400 nm. (d–f) Confocal one-photon fluorescent microscope images of vaterite particles loaded with CDs in the three different geometries: (d) spheres, (e) ellipsoids, and (f) toroids. The insets provide a closer view of a single particle with a scale bar of 0.5 μm. (g) Langmuir adsorption curve depicting the adsorption of CDs on a vaterite surface for different particle geometries. (h) Maximum adsorption of the CDs on vaterite for different geometries, as derived from the Langmuir equation.

Following these preliminary experiments, several in vitro experiments were conducted to demonstrate the imaging potential of CDs (see the Supporting Information for cell culturing and imaging details). Figure 4 shows confocal imaging of MDA-MB-231 incubated with 10 μM tetramethylrhodamine (TRITC) for 30 min and incubated for more than 2 h with 0.01 mg/mL CD–vaterite with different geometries. Imaging enabled visualization of red-stained cellular plasma and green CD–vaterite particles in the volume inside and outside the cell. Our results indicate distinct behaviors based on particle geometry. Elliptical particles were observed to penetrate extensively into the cell, as evident from bright fluorescence spots within the cell plasma. In contrast, spherical particles showed less penetration, although they were still detectable both inside and outside the cellular boundaries. Notably, toroidal particles exhibited brighter fluorescence, which suggests either enhanced interaction or accumulation at the imaging site. Cross-sectional analysis from the confocal images further confirmed that despite variations in fluorescence intensity and penetration, all particle shapes were effectively internalized by the cells. The internalization has been supported and verified using Z-stack. The fluorescent particles are visible as green emission in the insets of Figure 4a–c and are within the internal volume of the imaged cells. Further single-photon imaging of macrophage and glioma cells using CD–vaterite composites are shown in Figure S3 to demonstrate versatile imaging.

**Figure 4:**
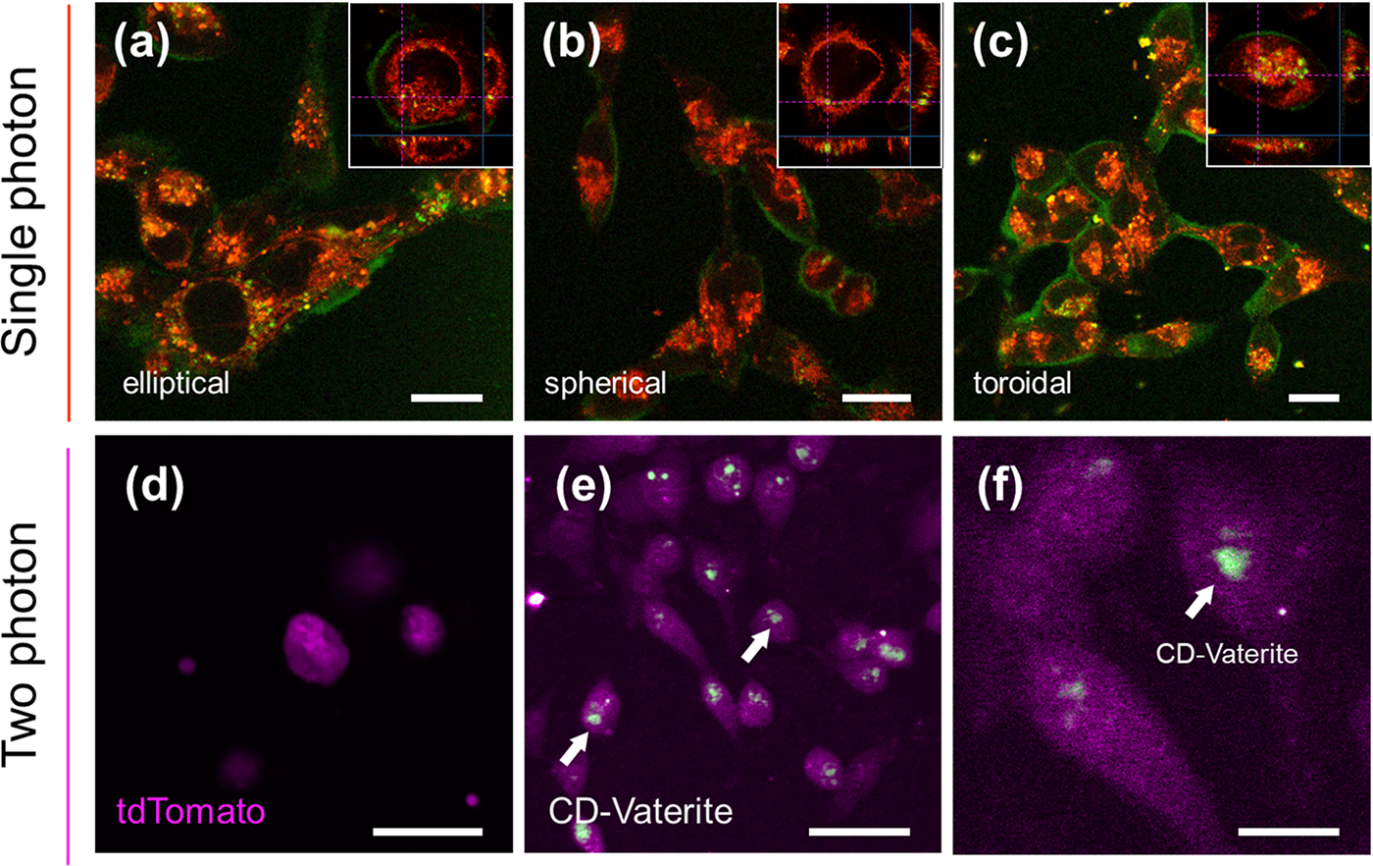
One-photon confocal images of breast cancer cells (MDA-MB-231) stained with TRITC in the plasma after incubation of 2 h with 0.01 mg/mL CD–vaterite: (a) elliptical, (b) spherical, and (c) toroidal. The inset is a cross-sectional element from the *x*–*y* plane. The scale bar corresponds to 20 μm. The orange emission is due to the TRITC staining, while the green emission is due to the CD–vaterite composites. (d) Two-photon confocal microscopy image of glioma cells stained with tdTomato. Scale bar = 10 μm. (e) Two-photon microscopy image of glioma cells with uptaken CD–vaterite composites. The green emission clearly shows the CD vaterite particles in the cells. Scale bar = 10 μm. (f) Magnified image of the glioma cells showing the green CD–vaterite emission. Scale bar = 3 μm.

The tdTomato-expressing glioma cells are of interest for potential tracking and understanding of future cancer treatments. Because of their accelerated metabolic activity, cancer cells uptake vaterite particles quite efficiently, as shown in previous studies.^37^Figure 4d displays the two-photon images of the bare cell without CD–vaterite composites. The fluorescent signal comes solely from tdTomato, which has a peak emission at 581 nm. Figure 4e,f presents the cells after the uptake of CD–vaterite composites. The CD–vaterite particles emit green light at 520 nm, thereby contrasting the red tdTomato fluorescence. This contrast allows for a clear visualization of the CD–vaterite composites within the cells. A magnified image of the excitation region is shown in Figure 4f where the vaterite particles inside the cell can be clearly seen. This detailed view further underscores the successful uptake of CD–vaterite particles by the C6-glioma cells and the effectiveness of two-photon microscopy in visualizing these particles.

Finally, particle visualization was tested in vivo. The process involved introducing spherical (Supplementary Figure S5) or elliptical (Figure 5) CD–vaterite composites to be injected into the bloodstream followed by two-photon microscopy through a cranial window (see the Supporting Information for animal testing and imaging details). The blood plasma was stained with Texas Red conjugated to a 70kDa dextran to facilitate the visualization of the blood vessels. The CD–vaterite particles are distinguishable from the background because of their green fluorescence emission. Individual vaterite particles flow relatively fast in the bloodstream in several images. For a more detailed observation, additional frames show the dynamic movement of these particles in the bloodstream and are provided in Supplementary Figure S4. Notably, there is also a green fluorescence observed, which can be attributed to the release of material from the vaterite particles. This study marks the first observation of vaterite particles in a mouse brain. The synergy between the fluorescence of CDs and the drug-carrying potential of vaterite emphasizes the promise of such composites in theranostic applications.

**Figure 5:**
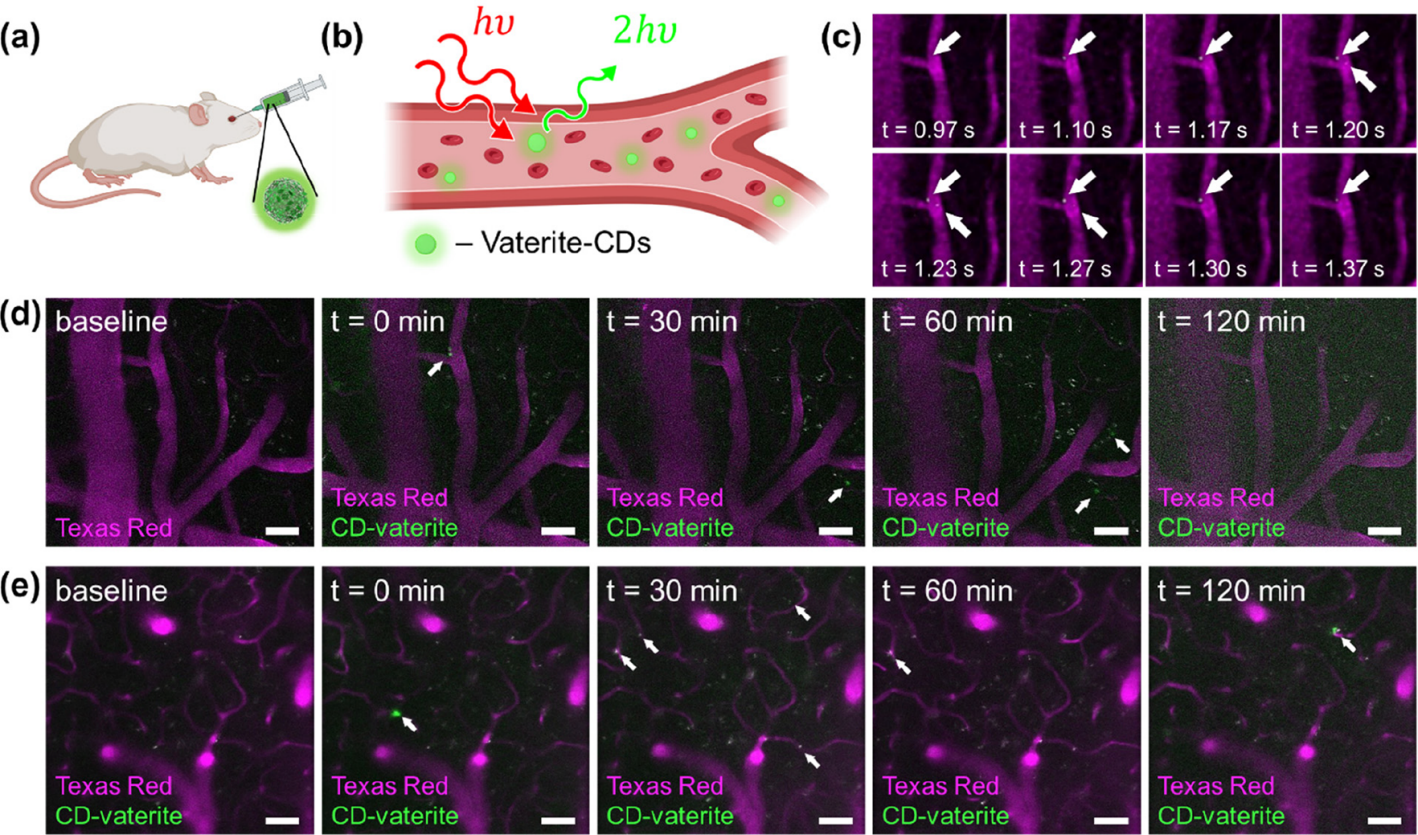
(a) In vivo imaging concept. (b) Render showing the two-photon fluorescence from CD–vaterite composites flowing in the bloodstream. (c) Stained blood vessels showing the movement on a single CD–vaterite composite over the course of ∼0.4 s. (d,e) Two-photon images at two different depths within the mouse cortex capture the presence of CD–vaterite composites in the bloodstream. The purple color corresponds to Texas Red employed to visualize the blood flow, while the green represents the CD–vaterite composites. White arrows highlight the CD–vaterite composites; it can be seen that at *T* = 30 min there are already CD–vaterite composites outside the blood vessels.

In this study, we have demonstrated a facile synthesis of *meta*-phenylenediamine CDs exhibiting robust optical fluorescence properties under both single-photon and two-photon absorption regimes. The detailed optical characterization of the CDs reveals a high two-photon cross-section across a wide range of pump wavelengths comparable with the well-established staining compound fluorescein. This underscores the potential of CDs as efficient and biocompatible nonlinear imaging agents. While other inorganic nonlinear materials, such as quantum dots, may have significantly higher fluorescent intensity and higher two-photon cross sections, CDs provide a biocompatible tool for imaging in cells and other organic materials. Biocompatibility plays a key role in the continuous imaging of cells both in vitro and in vivo. This may enable long-term experiments with minimal side effects and experimental deviation in cell studies. The CDs were loaded onto mesoporous vaterite nanoparticles in the next stage to demonstrate a complete biocompatible sample on pathways to a theranostic tool. The nonspecific binding of the vaterite surface to the CDs provides a straightforward CD loading route. Furthermore, this loading interaction could be further enhanced with specialized covalent linkers for specificity and is an outlook of this report.

The interaction of the CD–vaterite composites with MD-MDA-231 and C6-glioma cells was successfully investigated using one- and two-photon confocal microscopy. This was followed by injection and visualization of CD–vaterite composites in vivo in murine brain blood vessels for the first time. This allows for the tracking of particles in the bloodstream, thereby facilitating investigations involving blood vessels and the blood–brain barrier (BBB) and their disorders. Furthermore, with further optimization, this tool can allow real-time study of drug–cell interactions. Next, we aim to study the pharmacokinetics of the CD–vaterite complex through the accumulation of green fluorescence and determine the permeability of the BBB to vaterite formulations.

This work shows the powerful potential of CDs and vaterite as multifunctional tools that can be applied to both research and therapeutics.

## Supporting Information Available

Materials and CD–vaterite synthesis, structural analysis, linear and nonlinear optical characterization, optical properties, vaterite structural and loading properties, cell culture and imaging, animals and in vivo imaging, and in-brain images (PDF)
Video recording of in vivo imaging (AVI)

## Supplementary Material

nl4c00325_si_001.pdf

nl4c00325_si_002.avi
