# Supplementary material for: In-Brain Multiphoton Imaging of Vaterite Cargoes Loaded with Carbon Dots
Source: Nano Lett. 2024 May 23;24(27):8232–9. doi: 10.1021/acs.nanolett.4c00325 (PMC11247546; doi:10.1021/acs.nanolett.4c00325)
Supplement: Supplementary file 1 — nl4c00325_si_001.pdf [file nl4c00325_si_001.pdf]

Supporting Information

In Brain Multi-Photon Imaging of Vaterite Cargoes Loaded with Carbon Dots

Hani Barhum[1,2,3,*,=], Cormac McDonnell[1,3,=], Oleksii Peltek[4], David Kain[6] , Mariam Amer[2], Rudhvi Jain[5], Galit Elad-Sfadia[5], Muhammad Athamna[2,5] Pablo Blinder[5,6], and Pavel B. Ginzburg[1,3]

[1]Department of Electrical Engineering, Tel Aviv University, Ramat Aviv, Tel Aviv 69978, Israel [2]Triangle Regional Research and Development Center, Kfar Qara' 3007500, Israel; [3]Light-Matter Interaction Centre, Tel Aviv University, Tel Aviv 69978, Israel

[4]School of Physics and Engineering, ITMO University, St. Petersburg 191002, Russian Federation

[5]Neurobiology, Biochemistry and Biophysics School, Wise Life Science Faculty, Tel Aviv University, Tel Aviv 69978, Israel

[6]Sagol School of Neuroscience, Tel Aviv University, Tel Aviv 69978, Israel

*Corresponding author E-mail: hani.barhom@gmail.com
=equal contribution

S1 Materials

All the chemicals were purchased from Sigma Aldrich and were used without further purification. Meta-phenylenediamine (mPD) >99%, ethylene glycol 99.8%, hydrochloric acid 32% (HCl), acetonitrile (AcN) (AR), ethanol (AR), $CaCl_2$ 99.9%, Na_2CO_3 99.9%, $NaHCO_3$ 99.9%, poly-styrene sulfonate *70,000 Mw* (99.9%), fluorescein 98%, fluorescein isothiocyanate (FITC)>90%, MilliQ water, poly allylamine (PAH).

S2 CD synthesis

Fluorescent CDs nanoparticles were synthesized using meta phenylenediamine as the carbon source. Detailed information about CD synthesis appears in our previous publication[30]. Briefly, the solvothermal reaction was performed in an ethylene glycol medium and acidified by hydrochloric acid. 95 mL of ethylene glycol and 5 mL concentrated HCl solution in three-necked flasks were heated to 453^0K. Then 1 gram of meta phenylenediamine was added and refluxed for 3 hours. The dark solution is then cooled to room temperature. In the following step 1:5 ratio of carbonization extract and acetonitrile was mixed and centrifugation at 5 krpm for 30 min. The supernatant was transferred to a 100 mL beaker and then removed by heating to 353^0K. The remaining concentrated CDs dark solution was dried on a thin glass and then washed into a 100 mL beaker with ethanol. The solution was dried overnight in a vacuum chamber. The last steps of purifying were repeated to achieve pure CDs for analysis.

S3 Vaterite Synthesis

The vaterite synthesis was performed according to a previously developed protocol. Briefly, the general reaction conditions were 85:15 ethylene glycol to water ratio in a 40 mL Erlenmeyer, and the rotation speed of the magnet bar was set to 400 rpm at room temperature. CaCl2 0.025 M and Na2CO3 0.005 M concentrations were mixed for 120 min to achieve spherical particles. Elliptical particles were synthesized from $CaCl_2$ 0.005 M and $NaHCO_3$ 0.025 M*,* mixing for 120 min. Toroidal particles were synthesized with

the same spherical particle methodology synthesis conditions with the addition of 24 mg of polystyrene sulfonate before the addition of $CaCl_2$. The particles are collected using centrifugation at 5 krpm for 30 min. The particles were washed with EtOH 5 ml three times before use.

S4 Structural analysis

The size of the CD nanoparticles was examined using High-Resolution Transmission Electron Microscopy (HR-TEM) at an acceleration voltage of 200 kV. A Liquid Chromatography Mass Spectrometer (LCMS) was used to analyze the CD fragment mass. Fourier Transform Infrared spectroscopy (FTIR) was employed to extract the characteristic chemical groups of the CDs. Scanning Electron Microscopy (SEM) was employed to visualize vaterite particles.

S5 CD-Vaterite Composites

Purified and dried vaterite particles 20 mg were vortexed and sonicated for 5 mins every half hour with CD solutions of 2 mL at 2-4 mg/mL solution in EtOH. After two hours of adsorption, the solution was centrifuged at 15 000 rpm for 10 mins. The remaining CD solution was removed, and the particles were washed twice by centrifugation in EtOH or DIW, depending on the measurement requirements.

S6 Linear and Nonlinear Optical Characterization

Linear absorbance, emission, and photoluminescence excitation spectroscopy (PLE) were performed with a plate reader Synergy H1. Confocal microscopy images were obtained using a Leica SP8 system with 488 nm diode laser. Nonlinear fluorescence was investigated using an optical parametric oscillator (OPO) laser (Coherent Chameleon), with excitation wavelengths from 780 *to* 890 nm. The system generates 200 fs pulses at a repetition rate of 80 MHz. The laser output was focused into a cuvette holding the CDs with a microscope objective, and the reflected excitation was collected using the same objective. The reflected light was analyzed using a spectrometer after filtering the pump beam.

S7 Quantum Yield, Two-Photon Fluorescence Cross Section, and Two-Photon Absorbance

The Quantum Yield (QY) of CDs was measured in comparison to fluorescein and rhodamine 6G. The standard slope method was used. The obtained QY values are in the range ≈ 40 %, corresponding to the previously reported data[44]. To quantify the absorption cross-section and the multi-photon cross-section fluorescein dye molecules were used as a reference. The two-photon absorption coefficient was measured using the open aperture Z-scan technique. The cross-section equation is given by:

$$\delta = \delta_r \times \frac{\Phi_r}{\Phi} \times \frac{F\,C_r}{F_r\,C} \qquad (1)$$

where δ_r is the known fluorescein cross-section, Φ is CD QY, Φ_r is the relative QY used for fluorescein, F is the integrated fluorescence, n is the refractive index of the medium, and C is the molarity. The nonlinear absorption coefficient in the Z-scan technique is given by:

$$\Delta T(z) = \sum_{m=0}^{\infty} \frac{[-q_0(z,0)]^m}{(m+1)^{3/2}} q_0 = \frac{\beta I_0 L_{eff} z_0^2}{z^2+z_0^2} \quad (2)$$

where the nonlinear absorption $\beta = \frac{2\sqrt{2}}{I_0 L_{eff}} \Delta T$. T is the transmission from the sample, L_{eff} is the sample thickness, I_0 is the initial transmitted power far from the focal point.

S9 Vaterite Particle Characterization

CD-vaterite Loading Properties

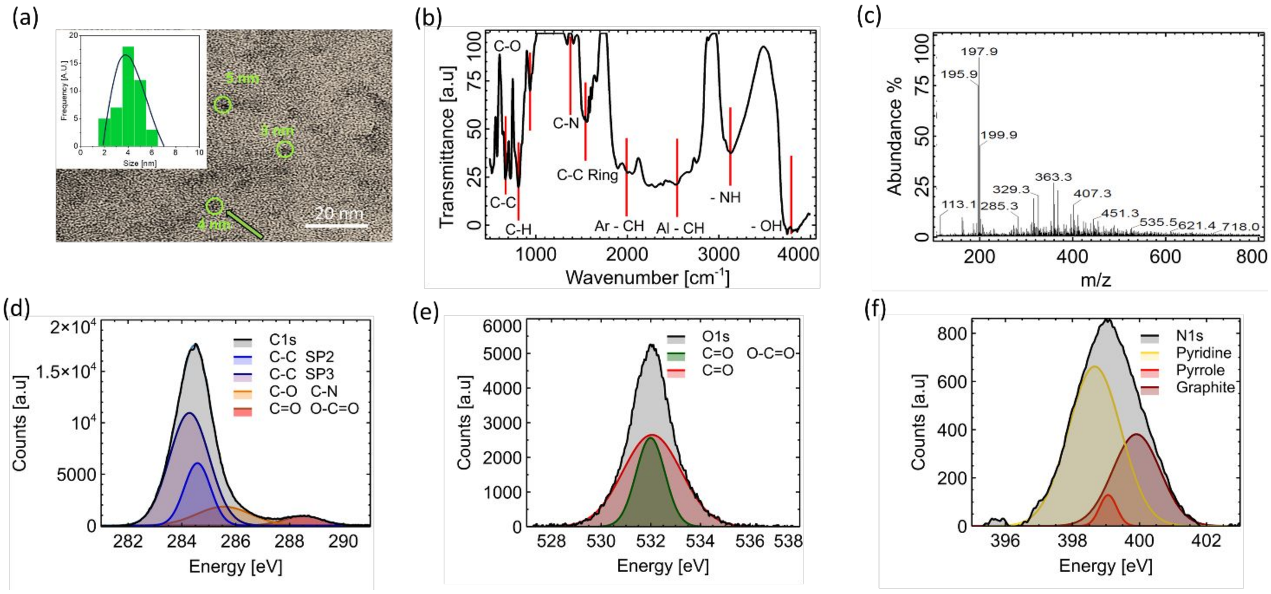

Figure S1. Comprehensive characterization of the synthesized CDs. (a) Transmission Electron Microscopy (TEM) image of the CDs, with the inset showing the size distribution of the CDs. (b) Fourier Transform Infra-Red (FTIR) spectroscopy of the CDs, with red lines indicating the chemical fingerprints of the CDs. The FTIR spectrum provides insights into the various functional groups present on the CDs. (c) Liquid Chromatography-Mass Spectrometry (LC-MS) of the CDs further confirms the CDs' chemical composition. (d) X-ray Photoelectron Spectroscopy (XPS) of C 1s, (e) O 1s, and (f) N 1s, revealing the presence of various functional groups on the CDs' surface.

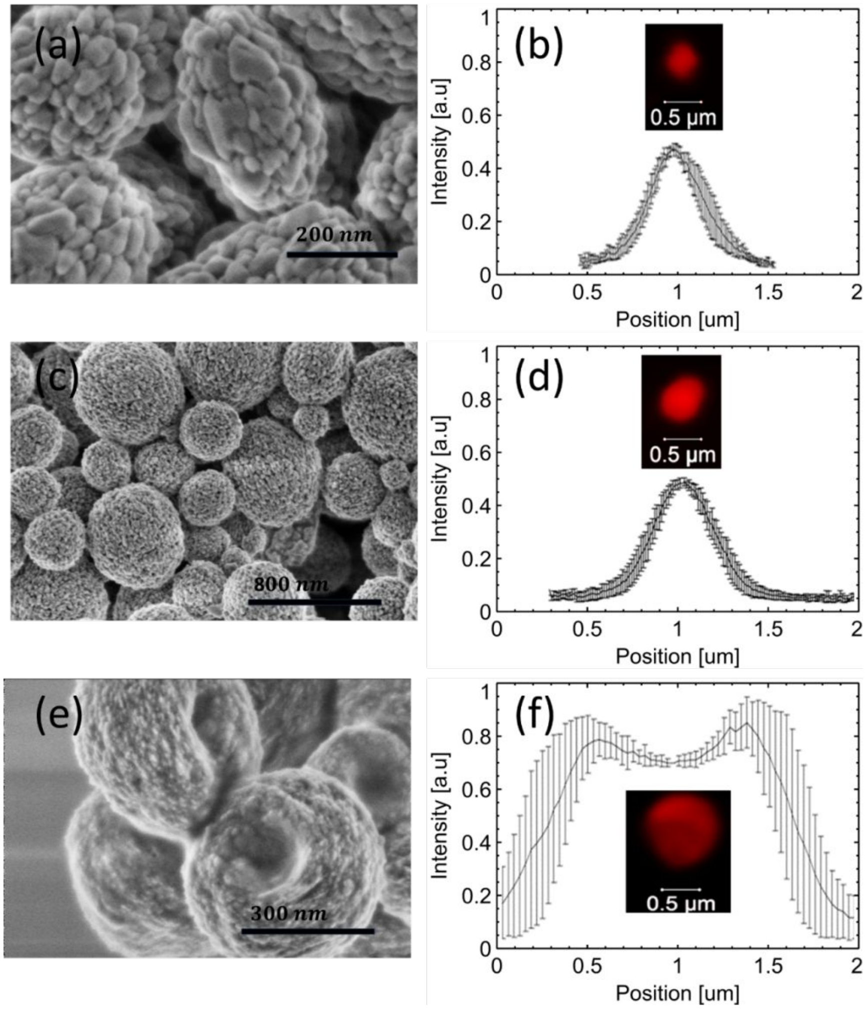

Figure S2. Characterization of vaterite particles and their fluorescent properties. (a) High-resolution SEM image of a pure vaterite particle. (b) SEM image of a vaterite particle post-fluorescent dye treatment, accompanied by an intensity profile showing the fluorescence distribution. The inset displays a confocal image of a spherical particle, highlighting its fluorescence. (c-d) Detailed images of spherical vaterite particles. (e-f) Detailed images of toroidal vaterite particles.

S10 Cell Culture and Imaging

The Glioma cells (GL261) were modified to constitutively express tdTomato. Cells were cultured in DMEM with 10% FBS and maintained at 37°C in a humidified 5% CO_2 incubator. Cells were plated in a 35 mm dish with a density of 5 X 105 cells/dish for 2 days until used in the imaging experiments. The cells were then loaded with the CD embedded in vaterite through incubation of 0.1 mg/mL for 2 hrs. Followed by washing and replacement with a cell growth medium. Imaging was conducted in an upright multiphoton imaging microscope (Sutter MOM, USA) equipped with a tunable laser source emitting 140 fs pulses at a repetition rate of 80 MHz (Chameleon Discovery NX laser, Coherent Inc). A 750 nm long-pass dichroic was used to direct fluorescent emitted photons into a collection arm consisting of a 535 ± 20 nm bandpass and a photomultiplier tube (Hamamatsu). The microscope was controlled through Scan Image (Vidrio Technologies Inc, USA).

Figure S3 presents comprehensive confocal and fluorescence imaging data. Panel (a) shows macrophage cells under 488 nm laser excitation, showing effective uptake of bare CDs. Panel (b) is a combined confocal and bright-field view of macrophages after incubation with both CDs and incubated with TRITC-stained vaterite particles under dual laser excitation. Panel (c) shows confocal imaging of C6-Glioma cells, which are embedded with CD-vaterite cargoes. The Glioma cells with their rapid metabolic activity showed efficient uptake of vaterite particles and are expressing td-tomato.

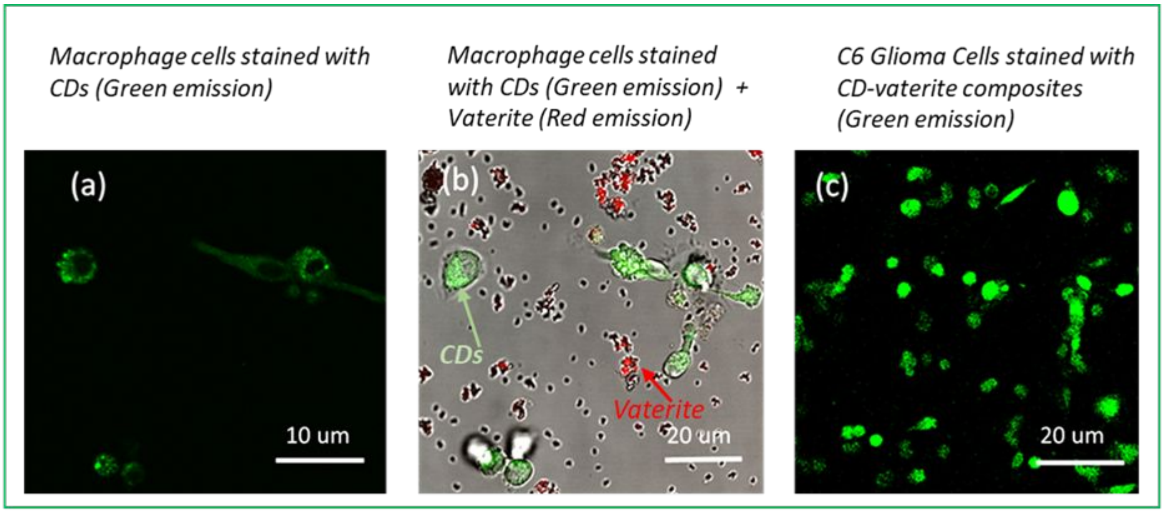

Figure S3. (a) Confocal microscope image of macrophage cells under 488 nm CW laser excitation with a 40x objective lens. (b) Combined confocal and bright field microscope image of Macrophage cells incubated with CDs under 488 nm CW laser excitation and Vaterite particles with RhodamineB isothiocyanate under 552 nm CW laser excitation with a 40x objective lens. (c) One-photon fluorescence confocal image of C6-Glioma cells embedded with a CD-vaterite composites.

S11 Animals and In Vivo Imaging

In vivo studies utilized healthy male C57BL/6J wild-type (WT) mice, aged 8 weeks and weighing 20-22 g, obtained from The Jackson Laboratory. The mice were housed in plastic cages within a specific pathogen-free environment maintained at 22 °C and a 40-60% humidity range. The mice were housed in a 12 h light/dark cycle and had ad libitum access to pellet food and water. The authorities of Tel Aviv University approved the experimental procedures for animal use and welfare and fully complied with IACUC guidelines.

The mouse was anesthetized using 5% inhalant isoflurane for induction and maintained at 1-2% in a 30/70 oxygen/N2O mixture throughout the surgical process. Following anesthesia, the mouse was placed onto a stereotaxic frame and the core body temperature was monitored and maintained at 37 °C using an electric heating pad. Ear bars and an incisor bar set suitable for a fat skull (3-4 mm) were used to secure the mouse. Before proceeding, eye lubricant was applied to prevent corneal desiccation. The mouse received an injection of dexamethasone (intramuscular, 0.2 mg/kg) to prevent swelling of the brain and/or inflammatory response and carprofen (subcutaneously, 5 mg/kg) for analgesia. Following these preparations, the skin surrounding the skull was cleaned using an isopropyl alcohol swab, and the scalp was cautiously removed and cleaned of any surrounding tissue or hair to expose the skull. The bone was

then slowly drilled with a high-speed manual drill (Osada, drill bit #5) equipped with a round engraver drill bit was used for the subsequent thinning of the skull for window preparation. The drilling process involved frequent pauses to allow for cooling of the skull bone using artificial cerebrospinal fluid (ACSF). With the aid of a compressed air duster, the skull piece was carefully detached from the skull, until the skull was transparent without any small debris. The thinned skull cranial was covered with #0 thickness cover glass for optical access. Then, a custom-made metal head frame was attached using cyanoacrylate glue (Loctite 401) and dental cement (high-Q-bond, BJM labs) to enable head fixation during imaging. To visualize the blood flow, mice were injected with 5% Texas Red dissolved in a CSF (25 µL, retro-orbitally), imaging was done in a number of plains across the cortex (baseline imaging). following baseline imaging 50ul of CD-Vaterite Composite was injected (retro-orbitally) and imaging was done at T=0,30,60,120 minutes after injection. Imaging was conducted using the microscope mentioned above at two wavelengths (920nm - Texas Red, 820nm- CD-Vaterite Composite).

S12 In-Brain Images

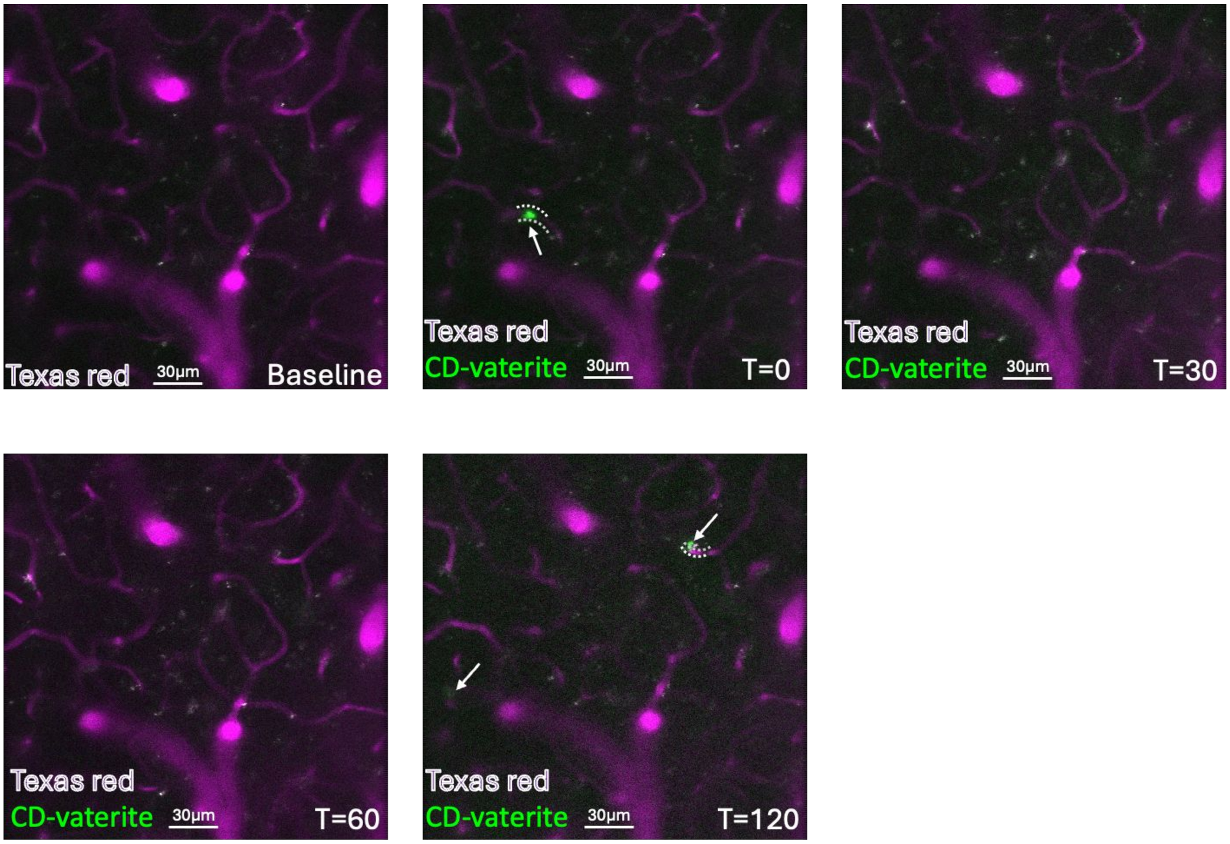

Figure S4. In vivo Two-Photon Imaging of CD-Vaterite Composites in Mouse Brain (a) A series of two-photon microscopy frames capturing the dynamic presence and movement of CD-vaterite composites in the mouse's bloodstream at different time points after injection (0,30,60,120min). The purple hue corresponds to Texas Red, utilized to delineate the blood flow, while the vibrant green signifies the CD-vaterite composites. White arrows pinpoint the CD-vaterite composites in transit. The scale bar is representative of 30 µm.

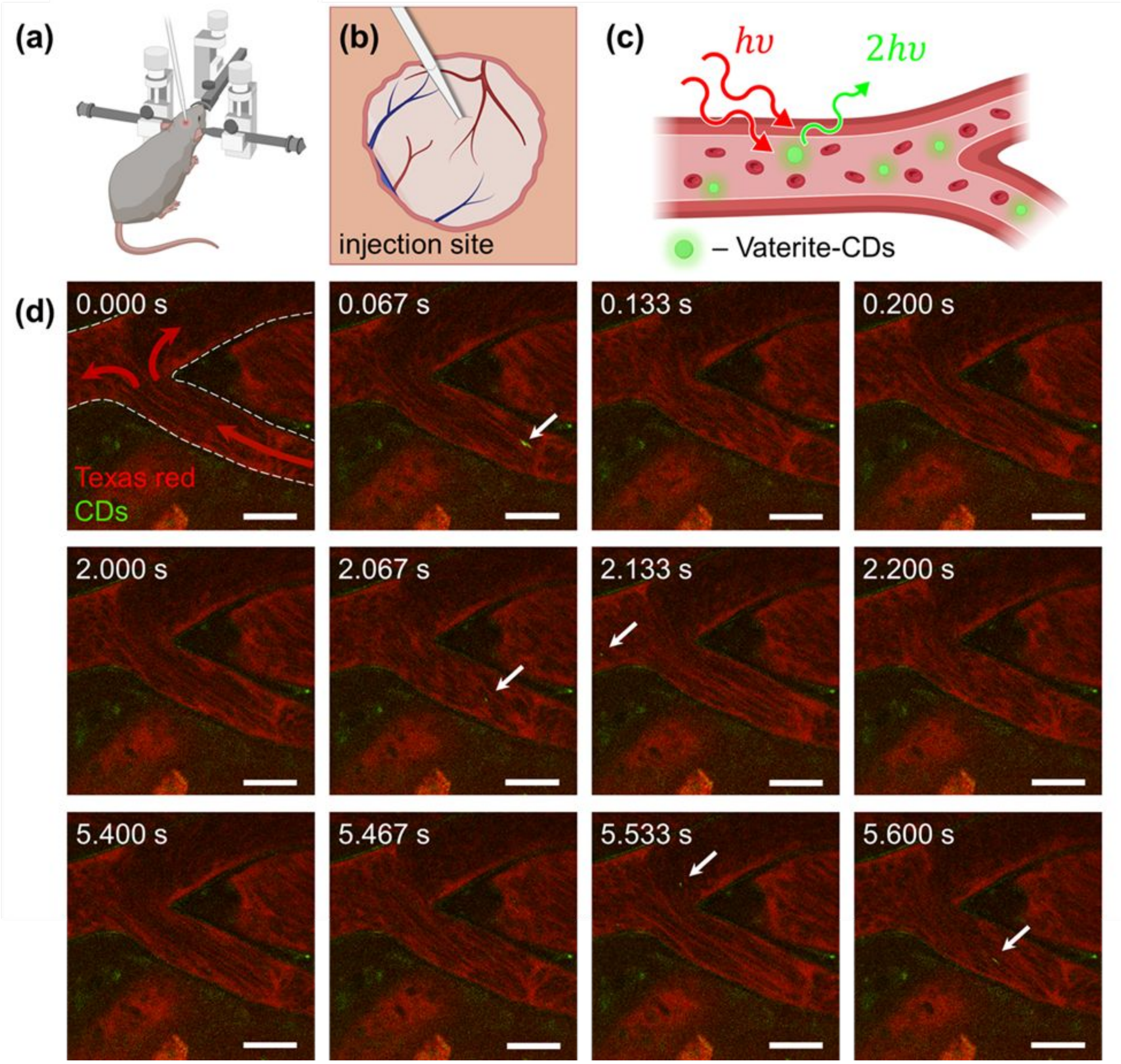

Figure S5. (a) Illustration of a mouse during the administration of CD-vaterite composites. (b) A schematic representation of the injection with CD-vaterite composites (c) A schematic of two-photon imaging used to visualize the CD-vaterite composites (d) Two-photon images capturing the presence of spherical CD-vaterite composites in the bloodstream. The red color corresponds to Texas Red, employed to visualize the blood flow, while the green color represents the CD-vaterite composites. The white dashed line outlines the monitored blood vessel border. Red arrows indicate the direction of the blood flow, while white arrows highlight the CD-vaterite composites. The scale bar corresponds to 30 µm. Figures (a)-(c) were created with BioRender.com.
